# Comparative study of the composition of cultivated, naturally grown *Cordyceps sinensis*, and stiff worms across different sampling years

**DOI:** 10.1371/journal.pone.0225750

**Published:** 2019-12-04

**Authors:** Yujue Zhou, Min Wang, Hui Zhang, Zhuo Huang, Jun Ma

**Affiliations:** College of Landscape Architecture, Sichuan Agricultural University, Chengdu, Sichuan, China; Institute for Biological Research, SERBIA

## Abstract

Natural *Cordyceps sinens*is, which is a valuable anti-tumor, immunomodulatory, and antiviral agent in Asia, has been overexploited in recent years. Therefore, it is important for cultivated *C*. *sinensis* to be recognized in the market. In this research, the main components of entirely cultivated, naturally grown *C*. *sinensis*, and stiff worms across different sampling years were detected and compared by HPLC-MS and UV spectrometry. The results indicated that the mean levels of adenosine and cordycepin were significantly higher, whereas the mean levels of mannitol and polysaccharides were remarkably lower in the cultivated type than in the natural type. No distinct difference in the average soluble protein content was observed. The composition of the stiff worms was similar to that of the natural herb, except that the total soluble protein content was higher, and that of mannitol was lower. In addition, the ultraviolet absorption spectroscopy of the three types showed high similarity at 260 nm. This research indicates that the main nutritional composition of cultivated and natural *C*. *sinensis* is identical and that cultivated type can be used as an effective substitute.

## Introduction

*Cordyceps sinensis* (Berkeley) Sacc. is a unique entomopathogenic fungus and valuable Chinese medicine resource that has been employed for treating various human conditions, including autoimmune disease, cancer, chronic inflammation, fatigue, and type II diabetes [1–3]. In winter, the fungus, mostly *Hirsutella sinensis* [4], parasitizes the ghost moth larvae (*Hepialus armoricanus* Obertheir, belonging to the order of Lepidoptera), and proliferates until the larva is converted into fungal hyphae; in summer, the stroma grows out of the dead caterpillar, leaving the exoskeleton intact (the fruiting body) [5]. Thus, this characteristic Chinese medicine is referred as ‘winter worm summer grass’ (Dong Chong Xia Cao in Chinese). The parasitic complex of the fungus and the caterpillar is mainly found in the soil of the prairie at an elevation of 3500–5000 m in Tibet, Qinghai, Sichuan, Yunnan provinces in China [6, 7]. Natural *C*. *sinensis*, whose availability is limited due to its extreme host range specificity and confined geographic distribution [8], has been overcollected to the brink of extinction. However, the technology for artificially breeding is not yet mature. There has been a massive disparity between supply and demand, resulting in skyrocketing prices in recent decades [9, 10]. The most common cultivated products on the market today are various health-care products consisting of fermentation liquid extracted from the mycelia of *C*. *sinensis* and other similar fungi [11]. However, due to the differences in the product form and the source of the effective components between cultivated and natural type, although the price of the cultivated type is lower than that of the natural one, it was not well accepted by consumers. On the other hand, the entirely cultivated type that not only morphologically resembles the wild one but also exhibits similar medicinal effects with controllable heavy-metal contamination necessitates more recognition by the market.

The quality assessment of *C*. *sinensis* is still in the preliminary stage [12]. The *Chinese Pharmacopoeia* specifies only the content of adenosine as a quality control marker [13]. Studies have shown that cordycepic acid (mannitol), cordycepin, and polysaccharides are also the main effective components [14, 15]. They are significant markers for the evaluation of the cultivated and natural type. Previous study shows that the nutritional value in terms of the levels of nucleosides, nucleotides, and adenosine is virtually the same between artificially and naturally cultivated samples [16, 17]. There is no difference in the chemical components detected between cultivated and natural Chinese *cordyceps* [18]. The extracts of both cultured and natural mycelia exhibit direct, potent antioxidant activities [19]. However, one study also reported that the contents of crude fat, total amino acids, and minerals were significantly different between natural and cultured samples [20]. Natural and cultured samples display significant differences in their metabolic profiles [21]. Thus, the available findings are confusing. Moreover, these reports have focused on fermentation extract or mycelia [22, 23]. A comprehensive exploration of the differences in the main components among different types by HPLC-MS and UV spectrometry has yet to be performed.

This study quantitatively and qualitatively described and compared the cordycepin, mannitol, adenosine, polysaccharides, and total soluble protein of entirely cultivated, naturally grown *C*. *sinensis* and stiff worms. It aims at providing useful information for people to understand the differences and accept the cultivated substitute more widely to reduce the use of the natural *C*. *sinensis*, an endangering species. This study also proves that the artificial cultivation of this precious herb is technically feasible.

## Methods

### Materials and instruments

#### Sample collection

**Fig 1:**
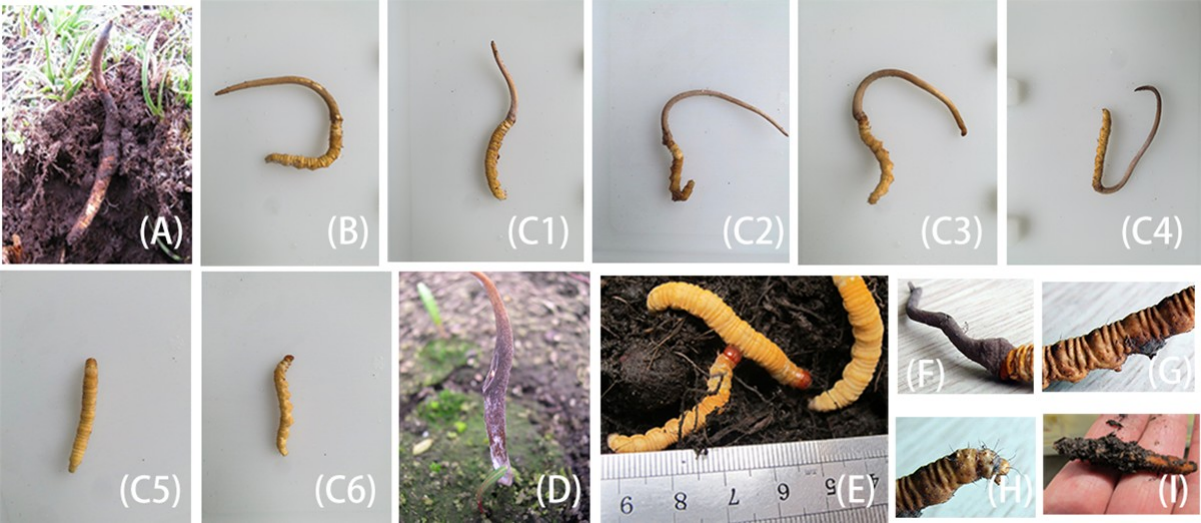
Photographs of the cultivated, naturally grown *Cordyceps sinensis*, and stiff worms across different sampling years. Note: A-C6: test samples; D: spore; E: ghost moth; F-H: details of cultivated *Cordyceps sinensis*.; I: stiff worm.

A total of 8 samples were divided into three types (Fig 1, Table 1). Entirely cultivated *C*. *sinensis* (B, C1-C4) was obtained through cultivation and inoculation by us. All the strains were the same *Hirsutella sinensis*, which were reserved separately for the next cultivation. The cultivation conditions of the strains and inoculated *C*. *sinensis* were also the same. Natural *C*. *sinensis* (A) was collected from the Pan’an village, Xiaojin County, Aba Tibetan and Qiang Autonomous Prefecture, Sichuan Province, China (102°1′-102°59′ E, 30°35′-31°43′ N), at an altitude of 3800–4500 m. The sampling site is publically owned. All the wild samples were acquired legally from the local people, and all the cultivated samples were bred by us, so we didn’t need any permits to carry out this study. The stiff worms were ghost moth larvae that were not able to grow fruiting bodies after artificial inoculation (C5, C6). Standard samples for cordycepin, mannitol, and adenosine were purchased from the China Food and Drug Testing Institute. We here state that we didn’t involve any endangered, threatened, or protected species or locations in this study.

**Table 1:** Experimental materials.

NO.	1(A)	2(B)	3(C1)	4(C2)	5(C3)	6(C4)	7(C5)	8(C6)
Type	Natural	Cultivated	Cultivated	Cultivated	Cultivated	Cultivated	Stiff worm	Stiff worm
Collection time	2018	2018	2016	2017	2015	2017	2017	2018

#### Sample processing

The samples were rinsed with deionized water, wrapped in filter paper and placed in an oven at 80°C for 24 hours. They were then pulverized at 6000 rpm for 3 min into fine powder and passed through a 60 mesh screen. From each sample, 0.25 g ± 0.005 g was weighed accurately and placed in a 50 mL volumetric flask, and 30 mL of Na_3_PO_4_ solution was then added at a concentration of 0.01 mol/L. The sealed bottle was shaken vigorously for 30 min and extracted in an ultrasonic extractor at a constant 60°C for 30 min. The extract was filtered through two-layer coarse filter paper, and the 50 mL filtrate was taken as the test solution.

#### Instruments

A Thermo Bio MATE 3S automated nucleic acid and protein analyzer and a Waters XEVO TQ mass spectrometer were used in this study. High-performance liquid chromatography (HPLC) was conducted with a Waters ACQUITY UPLC-I Class instrument. Thermo shaker was also used.

The chromatographic conditions were as follows: column: TSkgel ODS-100Z (3 μm, 4.6 mm×15 cm); column temperature: 50°C; flow rate: 0.8 mL/min; injection volume: 2 μl; solvents for the mobile phase: 10 mM aqueous ammonium acetate (A) and acetonitrile (B); gradient elution: 0 to 4 min of 5% B, 4 to 5 min of 40% B, 6 to 9 min of 95% B, 10 min of 5% B.

The mass spectrometry conditions were as follows: ion source: electrospray ionization (ESI) positive mode; scanning mode: multiple reaction ion detection (MRM); analytical temperature: 350°C; desolvated gas flow rate: (L/Hr): 700; capillary voltage: 3.3 KV. For ADE (adenosine), an ion pair of 268.22 > 136.07 was selected; for CORD (cordycepin), an ion pair of 252.22 > 136.07; and for MAN (mannitol), an ion pair of 183.14 > 69.03 (the former is the parent ion, and the latter is the most stable daughter ion) (Table 2).

**Table 2:** Optimized quantitative parameter conditions of the three standard samples.

Compound	Formula/Mass		Parent m/z	Cone Voltage	Daughters	Collision Energy	Ion Mode
Adenosine	267.00	1	268.22	22.00	136.07*	18.00	ES+
		2	268.22	22.00	119.09	44.00	ES+
Cordycepin	251.00	1	252.22	24.00	136.07*	16.00	ES+
		2	252.22	24.00	119.09	40.00	ES+
Mannitol	182.17	1	183.14	16.00	69.03*	12.00	ES+
		2	183.14	16.00	147.09	8.00	ES+

Note: The ion pairs with * in the table are the ion pairs used for quantification.

### Determination of main components

The three representative components of adenosine (C_10_H_13_N_5_O_4_), mannitol (C_6_H_14_O_6_), and cordycepin (C_10_H_13_N_5_O_3_) were measured by high-performance liquid chromatography-mass spectrometry (HPLC-MS).

Polysaccharides were determined by the sulfuric acid-phenol method [24]. The total soluble protein content was measured by placing the prepared test solution into the sample chamber of the fully automatic nucleic acid and protein analyzer to read the parameters after 10 seconds.

Each test sample was measured by a UV spectrometer at λ = 260 nm to compare the absorption spectra.

### Standard samples and standard curves

**Fig 2:**
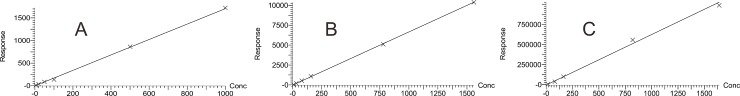
Standard curves of the three standard components. A: Adenosine: r = 0.997589, r2 = 0.995183, Calibration curve: 656.088 * x + 101.914; B: Cordycepin correlation coefficient: r = 0.999771, r2 = 0.999542, Calibration curve: 6.67068 * x + -12.245; C: Mannitol correlation coefficient: r = 0.997564, r2 = 0.995133, Calibration curve: 630.794 * x + -4898.04. The x-axis shows the substance concentration, and the y-axis shows the peak area.

The standard samples of cordycepin, mannitol, and adenosine were accurately weighed to obtain samples of 500, 250, 50, 25, 10, 5, and 2.5 mg, respectively, which were then dissolved in H_2_O. The solutions had a constant volume of 500 mL. A 1 mL aliquot of each sample was diluted again, and the volume was set as 1000 mL. Standard solutions with concentrations of 1000, 500, 100, 50, 20, 10, and 5 μg/L were prepared. After the HPLC-MS test, the standard curve of the concentrations and peak areas was obtained (Fig 2) as follows: adenosine: y = 656.088 * x + 101.914, r = 0.997589; cordycepin: y = 6.67068 * x—12.245, r = 0.999771; mannitol: y = 630.794 * x—4898.04, r = 0.997564, where x is the concentration, and y is the peak area.

### Data analysis

All data in the article were processed using SPSS25.0.

## Results

### HPLC-MS results

The HPLC-MS results showed that adenosine, mannitol, and cordycepin could be detected in cultivated *C*. *sinensis*. The peak times of all the samples were nearly the same, which indicated that the cultivated *C*. *sinensis* did not differ from the wild fungus in its main component types but that their concentrations varied.

#### Adenosine content

**Fig 3:**
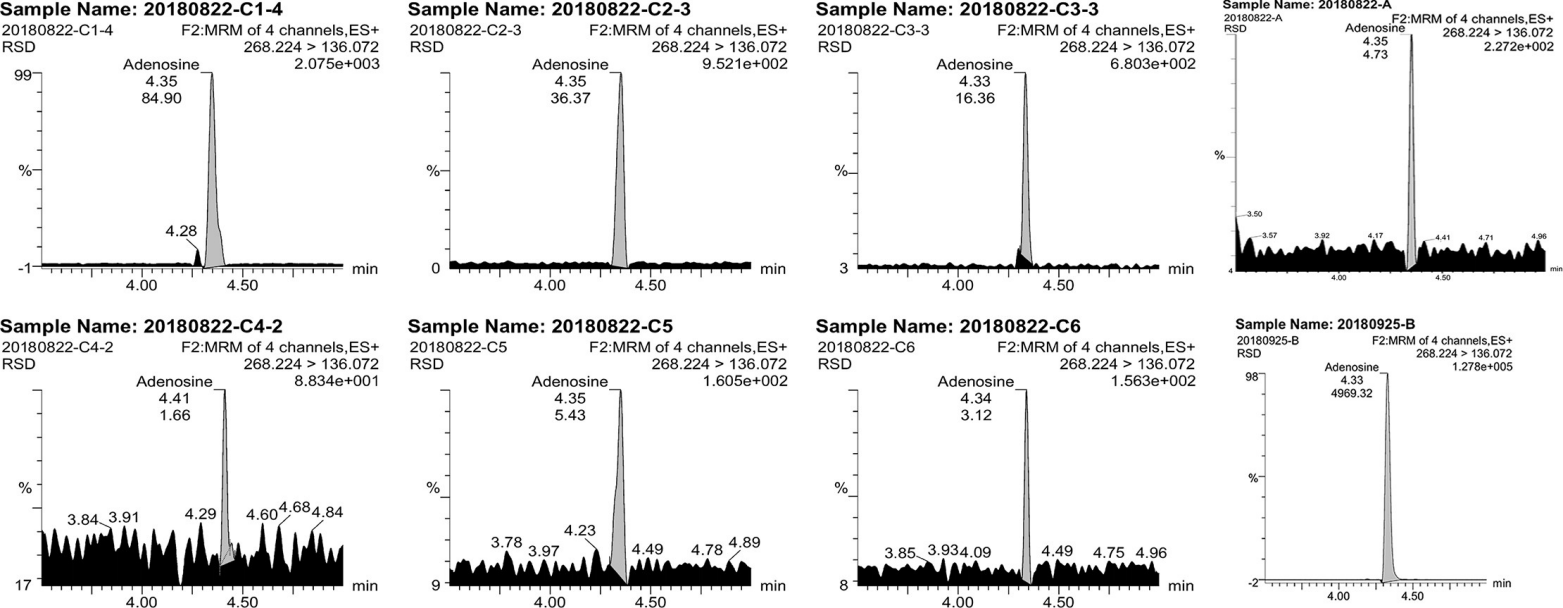
Adenosine contents in different samples.

Fig 3 illustrates that the peak time and retention time were basically the same in all the samples, showing no significant differences in adenosine. The maximum retention time of the test samples was 4.41 min (C4), and the minimum was 4.33 min (C3, B).

The adenosine contents of the eight samples varied from 1.2 μg/mL to 50.0 μg/mL, indicating that the adenosine contents of samples from different sampling years and of different types were significantly different (Table 3). The adenosine content of the cultivated type harvested in 2018 (B, 8.05 μg/mL) was significantly higher than that of the wild type (A, 3.00 μg/mL) in the same year (P<0.01). The adenosine contents of cultivated *C*. *sinensis* of different quality also exhibited extreme differences (P<0.01): the content of C1 (in which the worm body was filled, and the fruiting body was short and robust) was 50.0 μg/mL, whereas that of C4 (in which the appearance of the worm body was dry, and the fruiting body was thin and long) was 1.20 μg/mL. The mean total adenosine content of cultivated *C*. *sinensis* harvested from 2015 to 2018 (B, C1-4, 18.13 μg/mL) was significantly higher than that of the natural type (A, 3.0 μg/mL). There was no significant difference in adenosine content between the stiff worms (C5: 3.40 μg/mL, C6: 2.10 μg/mL) and natural type (P>0.05).

**Table 3:** Contents of the main components of all *C*. *sinensis* samples (μg /mL).

Sample No	Adenosine		Cordycepin		Mannitol		Polysaccharides		Total Soluble protein content	
	Concentration	Content %	Concentration	Content %	Concentration	Content %	Concentration	Content %	Concentration	Content %
A (1)	3.00^bAB^±0.12	0.06	3.00^cdBCD^±0.14	0.06	502.10^eE^±8.38	10.04	228.23^fE^±4.14	4.56	3.97^dC^±0.15	0.08
B (2)	8.05^cC^±0.23	0.16	22.10^fF^±0.22	0.44	562.30^fF^±8.09	11.25	286.63^gF^±5.18	5.72	4.57^fE^±0.13	0.09
C1(3)	50.00^fE^±0.13	1.00	6.00^eE^±0.23	0.12	578.30^fF^±9.23	11.57	167.35^cdCD^±3.53	3.32	3.43^aA^±0.19	0.07
C2(4)	21.60^eD^±0.17	0.43	3.10^dCD^±0.19	0.06	128.10^bB^±3.22	2.56	159.57^cBC^ ±3.16	3.08	3.61^bB^±0.14	0.07
C3(5)	9.80^dC^±0.11	0.20	3.20^dD^±0.15	0.06	405.20^dD^±5.27	8.10	126.30^aA^±2.19	2.53	3.35^aA^±0.15	0.07
C4(6)	1.20^aA^±0.13	0.02	2.40^aA^±0.25	0.05	514.00^eE^±6.25	10.28	146.17^bB^±2.24	2.93	3.76^cB^±0.20	0.08
C5(7)	3.40^bB^±0.17	0.07	2.70^bAB^±0.14	0.05	321.80^cC^±2.19	6.44	179.00^deD^±3.26	3.56	4.51^fE^±0.17	0.09
C6(8)	2.10^abAB^±0.19	0.04	2.80^bcBC^±0.16	0.06	0.60^aA^±0.17	0.01	185.20^eD^±3.89	3.71	4.21^eD^±0.12	0.08

Note: The same letter in each column represents no difference according to Duncan's method; lowercase letters indicate significant differences, P<0.05; and capital letters indicate extremely significant differences, P<0.01.

#### Cordycepin contents

**Fig 4:**
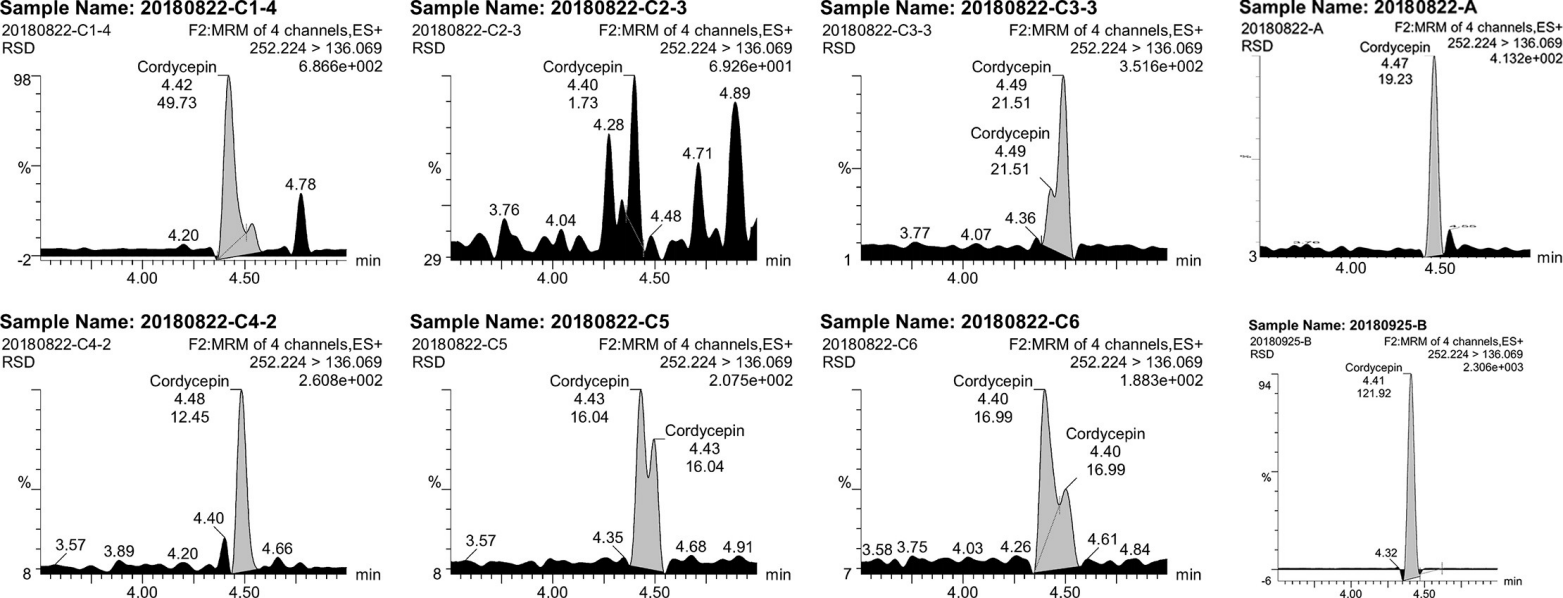
Cordycepin contents in different samples.

Fig 4 shows that the average peak time of cordycepin was approximately 4.5 min, and the peak time and retention time were longer than those of adenosine and mannitol. The stiff worms (C5, C6) were prone to exhibit close twin peaks where the second peak was notably lower than the first peak.

The cordycepin content ranged from 2.4 μg/mL to 22.0 μg/mL and fluctuated less than the adenosine content (Table 3). However, the data indicated a significant difference in cordycepin content in *C*. *sinensis* samples from different sampling years and of different types, even within the same year. The cordycepin contents of the cultivated (B, 22.10 μg/mL) and wild (A, 3.00 μg/mL) types in 2018 were extremely significantly different (P<0.01). The average cordycepin content of cultivated *C*. *sinensis* from different years was 7.36 μg/mL, which was markedly higher than corresponding value of 3.0 μg/mL for natural type. The cordycepin contents of the stiff worms produced in 2017 and 2018 (C5 and C6) were 2.7 μg/mL and 2.8 μg/mL, respectively, and this difference was not significant (P<0.05).

#### Mannitol content

**Fig 5:**
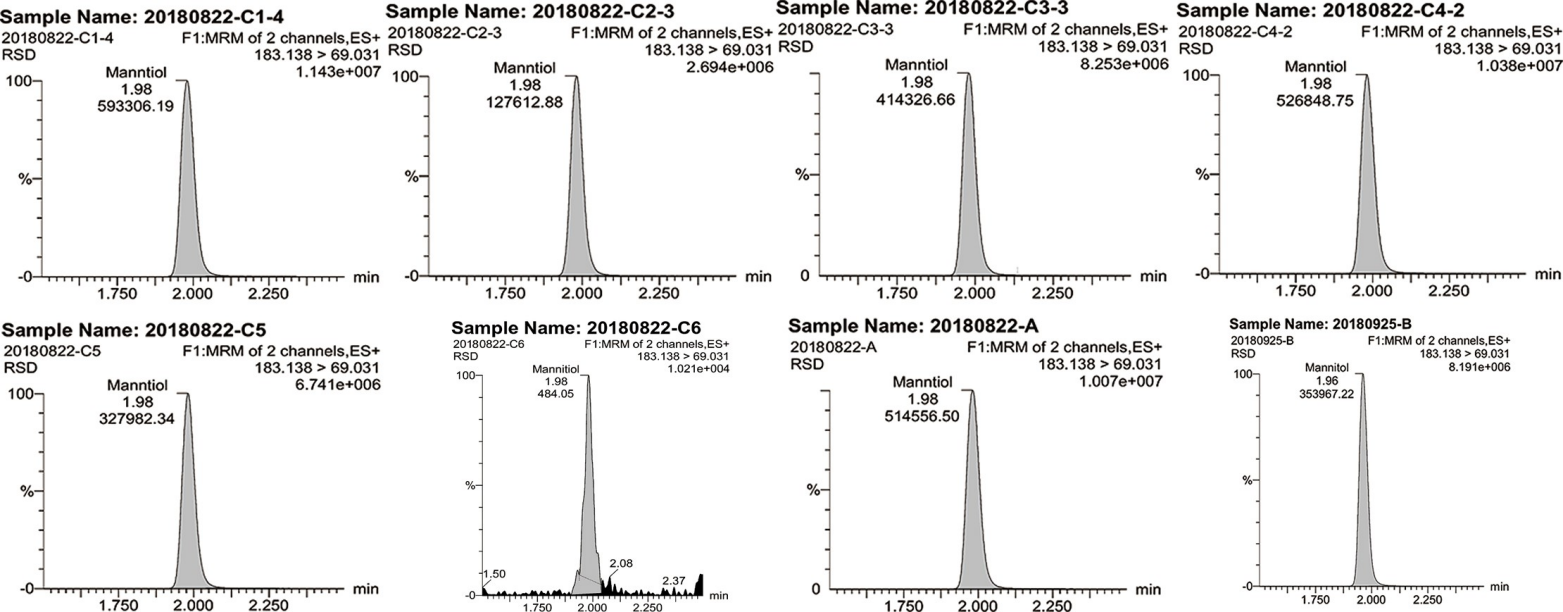
Mannitol contents in different samples.

The mannitol test results showed a similar peak time and retention time of all the samples, while the contents differed significantly (Fig 5). The differences in the mannitol contents were the largest among all of the indicators tested.

The measured concentration of mannitol was the highest among all indicators, indicating that mannitol was one of the major components. It also presented the greatest range (Table 3) of 0.60 μg/mL (C6) to 578.30 μg/mL (C1) compared with other indicators. The mannitol contents of *C*. *sinensis* from different sampling years varied remarkably from 128.10 μg/mL (C2, 2017) to 578.30 μg/mL (C1, 2016). The mannitol contents of cultivated *C*. *sinensis* harvested in the same year (2017) with different quality levels also showed significant differences (P<0.01), ranging from 128.10 μg/mL (C2) to 514.0 μg/mL (C4). The mannitol concentrations of the cultivated type ranged from 128.10 to 578.30 μg/mL with an average content of 437.58 μg/mL, which was notably lower than those of the natural type (502.10 μg/mL). The mannitol concentration was significantly different in stiff worms from different years (321.80 μg/mL (C5) versus 0.60 μg/mL (C6)), and the average mannitol level of the stiff worms (161.2 μg/mL) was notably lower than those of the wild and cultivated types.

Overall, the HPLC-MS results demonstrated that all of the main effective substances, including adenosine, cordycepin, and mannitol, could be detected in cultivated type and that the peak times were similar to those of the natural type. The only difference between cultivated and natural *C*. *sinensis* was in the concentrations detected. This analysis indicates that the components of the cultivated *C*. *sinensis* and the wild type are virtually identical.

### Assessment of polysaccharides

The results of analysis by the sulfuric acid-phenol method showed that the contents of polysaccharides differed between samples, with the distribution ranging from 126.30 μg/mL to 286.63 μg/mL. The polysaccharide content of cultivated *C*. *sinensis* (B, 2018) was 286.63 μg/mL, which was significantly higher than that of (P<0.01) the natural type (A, 228.23 μg/mL). However, the average polysaccharide content of cultivated *C*. *sinensis* was 177.20 μg/mL, which was significantly lower than that of the natural polysaccharides (228.23 μg/mL) (P<0.01). The average content of polysaccharides in the stiff worm was 182.1 μg/mL, which was also significantly lower than that of wild type.

### Assessment of total soluble proteins

The data revealed that soluble proteins were not the main component (3.35–4.57 μg/mL). The soluble protein content of the cultivated type (B, 2018) was 4.57 μg/mL, whereas the content of natural one from the same year (A, 2018) was 3.97 μg/mL, and the difference was significant (P<0.01). The soluble protein contents of cultivated *C*. *sinensis* from different sampling years were significantly different (P<0.01). The average soluble protein content of the stiff worms was 4.36 μg/mL, which was significantly higher than those of the cultivated type (3.74 μg/mL) and the wild type (3.97 μg/mL) (P<0.01).

### Ultraviolet absorption spectrum results (λ = 260nm)

**Fig 6:**
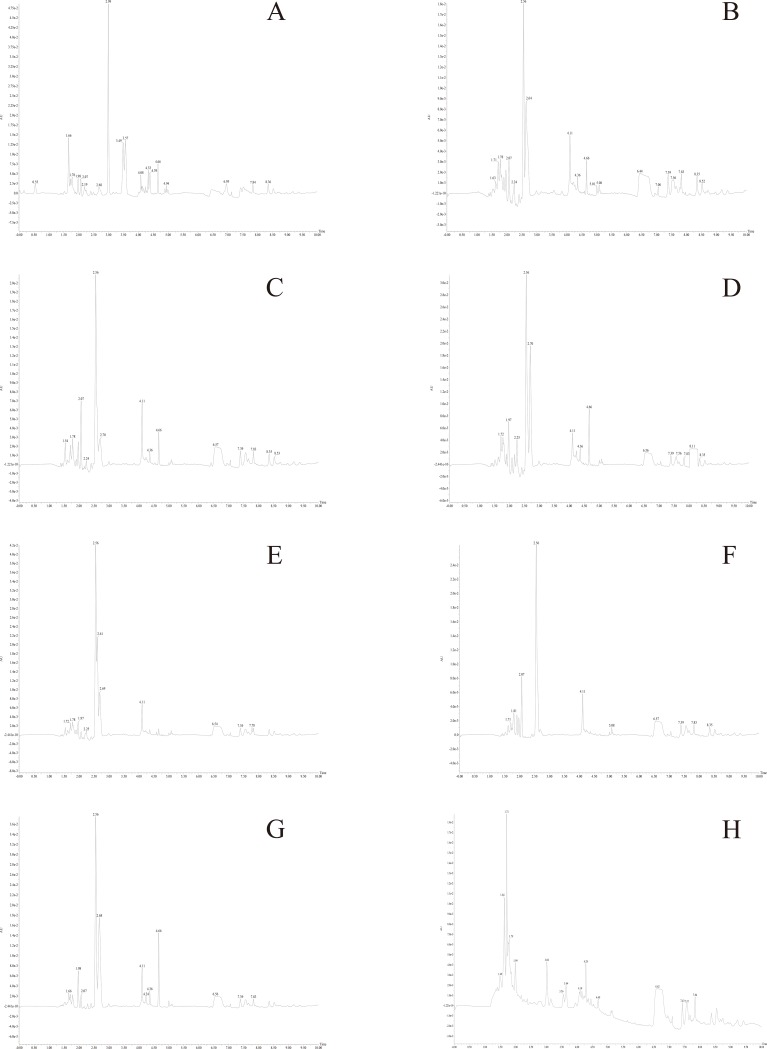
Ultraviolet absorption spectroscopy of all the samples.

As shown in Fig 6, the UV absorption spectrum of each sample was almost the same, but Fig 6A, Fig 6B, Fig 6C, and Fig 6D (cultivated *C*. *sinensis*) reveal a similar pattern in which the central peak is intense, and there are several consecutive small peaks around the central peak. The UV profiles of Fig 6E and Fig 6F (both were stiff worms) could be classified into one type in which the central peak is followed by a small peak before eventually leveling out. The UV absorption spectra of Fig 6G (A, natural *C*. *sinensis*) and Fig 6H (B, cultivated *C*. *sinensis*) are significantly different. The maximum absorption peak of Fig 6H is earlier than that of Fig 6G and other samples, and the numbers and contents of the other absorption peaks are more complicated than those of Fig 6G.

### General evaluation

Comparison of the mean values of various component indicators of cultivated *C*. *sinensis* and stiff worms obtained from 2015–2018 with those of the natural type in 2018 (Table 4) revealed distinct differences in the indicators. However, with further analysis, we could see that the levels of small molecules such as adenosine and cordycepin were significantly higher in cultivated *C*. *sinensis* than in the wild type. Although there were differences between the stiff worms and the natural type, the absolute difference was not significant. The content of polysaccharides was significantly different between the three types in the following order (P<0.01): wild type> stiff worm> cultivated type. Soluble protein was not the main component of *C*. *sinensis* (below 0.01%), suggesting no significant difference between the natural and cultivated types (P>0.05), but the content of this component was significantly different from that in stiff worms (P<0.01), with the following order being observed: stiff worm> cultivated type> natural type.

**Table 4:** General comparison of the contents of the main components of three types of *C*. *sinensis* (μg/mL).

Sample No	Adenosine		Cordycepin		Mannitol		Polysaccharides		Total soluble protein content	
	Concentration	Content %	Concentration	Content %	Concentration	Content %	Concentration	Content %	Concentration	Content %
Wild type	3.00^bB^±0.13	0.06	3.00 ^bB^±0.29	0.06	502.10 ^cC^±9.11	10.04	227.80 ^cC^±4.24	4.56	3.97 ^aA^±0.16	0.08
Cultivated type	18.13^cC^±0.22	0.36	7.36 ^cC^±0.14	0.15	437.58 ^bB^±8.12	8.75	175.85 ^aA^ ±3.88	3.51	3.7443 ^aA^ ±0.26	0.07
Stiff worm	2.75^aA^±0.19	0.05	2.75 ^aA^±0.16	0.05	161.2 ^aA^±2.28	3.22	181.75 ^bB^ ±3.20	3.63	4.3565 ^bB^ ±0.14	0.08

Note: The same letter in each column indicates no difference by Duncan's method; lowercase letters indicate significant differences, P<0.05; and capital letters indicate extremely significant differences, P<0.01.

## Discussion

*Cordyceps sinensis* has gained public popularity and global scientific attention due to its wide range of nutritive and medicinal properties. However, the natural *C*. *sinensis* has been excessively harvested in the last two decades, leading to a drastic decrease in wild populations. Hence, this study attempts to investigate the differences in the entirely cultivated, wild type, and stiff worms by identifying and comparing the major composition. It aims at providing useful information to understand the differences and encouraging the use of cultivated alternatives. The results indicate that all the characteristic components of the natural herb can be detected in the cultivated type and stiff worms, but the concentrations vary among different types and types from different yielding years. Our results are in some respects similar to those in previous studies comparing the constituents in the cultivated, natural *C*. *sinensis* and related species [20, 23], but in the present study, the representative composition has been comprehensively compared by employing the artificially breeding *C*. *sinensis* across different years that has the same appearance of the natural type instead of fermentation extract or mycelia (Fig 1).

Adenosine, cordycepin, cordycepic acid (mannitol), and polysaccharides are four major and effective components of *C*. *sinensis*. Among these components, adenosine has been used as a premier marker for quality control of *C*. *sinensi*s [13] and is well known to depress the excitability of CNS neurons, to inhibit the release of various neurotransmitters presynaptically and to possess anticonvulsant activity [7]. The molecular structure of cordycepin is CsHyON, which is essentially a derivative of adenosine. Cordycepin is the first nucleoside antibiotic isolated from *Cordyceps militaris*, a species related to *C*. *sinensis* that is commonly used as a substitute [25]. Whether or not natural and cultured *C*. *sinensis* contain cordycepin is still controversial [26–29], but cordycepin contained in natural and cultivated type is confirmed in this and other reports [30, 31]. Adenosine and its derivatives, including cordycepin, are very useful due to their powerful bactericidal, antiviral, fungicidal, and anticancer functions, presenting strong pharmacological and therapeutic potential to cure many dreadful diseases [32]. This study showed that the contents of adenosine-related substances (adenosine and cordycepin) in cultivated *C*. *sinensis* were significantly higher than in the natural type, whereas the contents in the stiff worms were not significantly different from those in natural ones. The results echo the previous studies that the contents of nucleosides (cordycepin, adenosine, etc) from cultured Cordyceps were higher than that of those from wildlife [29, 31], and that the levels of adenine and adenosine in the cultured sample are considerably higher than the natural ones [20, 29]. One study also states that the amount of nucleosides, especially adenosine in cultured *C*. *sinensis* is higher than that in natural type, and cultured *C*. *militaris* exhibits much higher content of cordycepin [33]. Chassy and Suhadolnik report on the biosynthesis of cordycepin in *C*. *militaris* by radioimmunoassay [34], from which we can speculate that higher adenosine and cordycepin in the cultured type might be induced by the favorable controlled lighting, moisture, and temperature during the initial period of asexual reproduction, which facilitate the absorption and transportation of the adenosine and cordycepin.

Cordycepic acid, also known as mannitol, is mainly used as an anhydride and diuretic in medical treatment. It has pharmacological effects such as increasing plasma osmotic pressure, anti-tussive, anti-free radical activities [7], and cerebrovascular dilation [35]. It can be employed in the treatment of meningioma as a liquid chemoembolization agent [36], in the treatment of intracerebral hemorrhage [37], and for downregulating intracranial pressure [38]. In the present study, there was a significant difference in the contents of mannitol among the three types of specimens in the following order: natural type > cultivated type > stiff worm, but the difference between the absolute values of the last two specimen types was not significant. Previous study exhibits consistent findings that natural herb contains more free mannitol and a small amount of glucose, while mannitol in cultured *C*. *sinensis* and cultured *C*. *militaris* is much less and free glucose is only detected in a few samples [39]. Additionally, natural products have a significantly higher content of mannitol compared with the submerged cultured mycelia [40]. Adenosine, cordycepin, and mannitol are small molecules that are the primary molecules constituting nucleic acids and polysaccharides. From the perspective of molecular structure, mannitol is the reduction product of mannose, which is the C-2 epimer of glucose [41]. Therefore, the discrepancy in the content of mannitol might have been caused by the different transformation processes and equilibrium sites associated with varying transformation efficiencies under different biological conditions. However, the specific transformation processes of the three compounds in the ghost moth body and *C*. *sinensis* body are still poorly understood, which necessitates further research.

Polysaccharides are one of the most abundant components of *C*. *sinensis* [42]. Since Miyazaki first obtained water-soluble polysaccharides from *C*. *sinensis* fruiting bodies [43], researchers have conducted extensive research on the functions of *C*. *sinensis* polysaccharides. Studies have demonstrated the pharmacological use of *C*. *sinensis* polysaccharides to achieve immunostimulation, antitumor activity, and free radical scavenging [44]. A more recent study finds that there are relatively high similarities among the polysaccharides from different batches of cultivated *C*. *militaris*, and also between the polysaccharides from cultivated *C*. *militaris* and natural *C*. *sinensis* [45]. Polysaccharide is significantly higher for cultured than those of natural samples [20]. Conversely, in our research, noticeable higher contents were detected in the natural herb. The results of this study indicated significant differences in the following order: natural type > stiff worm> cultivated type. The reasons for these findings can be twofold. First, there are differences in the structure of animal tissues and fungal tissues, and the main dry matter component of animal tissues is protein, whereas the main component of fungi is polysaccharides. When ghost moth larvae are transformed by the fungus after successful inoculation, the original animal tissue components are turned into fungal hyphae. Therefore, the polysaccharide contents of both the cultivated type and the stiff worm were significantly lower. Second, notable differences are found in the diets of wild ghost moths and artificially reared ghost moths. Wild ghost moth larvae are omnivorous insects that feed on varied diets, including plant underground roots and soil humus [46]. A wide variety of food sources (generally more than ten species) are available to wild ghost moths. In contrast, the diet of the artificially cultivated ghost moths is usually limited to 1–2 species. As a result of this simplified diet, the monosaccharides available to synthesize polysaccharides are markedly less abundant than in wild ghost moths. Therefore, *C*. *sinensis* might be affected by different cultivation conditions and harvesting time, resulting in different polysaccharide contents, which implies that the originality and growth environment can considerably affect the chemical composition of *C*. *sinensis*. This is in line with the previous studies that among different habitats [47] and different harvesting time, the contents of various components of *C*. *sinensis* differ significantly [48]. Limited by the structural diversity and complexity of polysaccharide molecules and current research methods, the structure of the polysaccharides in *C*. *sinensis* is currently inconclusive, which remains to be further studied [49].

Proteins in *C*. *sinensis* play role in biological processes such as ribosome formation, stress adaptation for temperature reduction and cell cycle control [50], which is not the main effective component. Previous researches report on the proteomic analysis of *C*. *sinensis* to determine the proteins [50] and provide basic proteome profile for further study [16]. However, very limited studies are available for the comparison of the soluble protein in the natural and cultivated *C*. *sinensis*. In this study, the soluble protein content of the stiff worms was significantly higher than those of the natural type and cultivated type (P<0.01), but no significant difference was detected. This could be explained by the fact that less effective transformation of animal tissues into fungal tissues drives the protein contents higher in the cultivated type than those of the natural type. Likewise, a large number of animal components (insect proteins, glycolipids) in the original stiff worm body were not effectively transformed due to failed fruiting body formation, resulting in a higher soluble protein content in the cultivated type. Additionally, ghost moth larvae have a life cycle of up to 5 years, most of which is spent underground [51]. By comparison, the life span of artificially reared ghost moths is compressed to as little as one year due to the beneficial controlled environment. Thus, rapid growth leads to a shortened productive nutrition accumulation period. Therefore, the various abundant nutrient resources from food and long growth time of the wild ghost moth guarantee the synthesis of various proteins in its body and the effective accumulation of various dry matter components.

In the limitation of this study, in order to dehydrate the fresh samples as soon as possible to avoid bacterial, viral, and insect contaminants, the preparation of the test solution for soluble protein test was set at a higher temperature, which might lower the soluble protein tested, however, it could be offset at some extent by the following ultrasonic extractor that could break the macromolecule. Besides, although the major components have been identified and compared in the natural and cultivated *C*. *sinensis*, extensive work is still needed to define the transformation processes and the exact roles of these components.

## Conclusions

This study compared the main components of wholly cultivated, natural *C*. *sinensis*, and stiff worms. The test results showed that all five examined effective components of natural *C*. *sinensis* could be detected in cultivated type. More importantly, the contents of adenosine and cordycepin were even higher in the cultivated type. Additionally, the adenosine content of cultivated type in different years exceeded 0.01%, meeting the quality control requirements specified in the *Chinese Pharmacopoeia (2015 version)* [13]. Although the contents of cultivated *C*. *sinensis* were inconsistent, showing remarkable differences in cultivated type from different years, we can conclude that cultivated *C*. *sinensis* could be used as a reliable substitute of the natural herb for mass production of the medicinal fungal materials.

## Supporting information

S1 Fig Results of ultraviolet absorption spectroscopy under 260nm. (RAR)

S1 File Results of the first test of adenosine. (RAR)

S2 File Results of second test of adenosine (Sample B retested). (RAR)

S3 File Results of the first test of cordycepin. (RAR)

S4 File Results of the second of cordycepin (Sample B retested). (RAR)

S5 File Results of the first test of mannitol-1. (RAR)

S6 File Results of the first test of mannitol-2. (RAR)

S7 File Results of the second test of mannitol (Sample B retested). (RAR)
